# Congenital Cytomegalovirus Infection Burden and Epidemiologic Risk Factors in Countries With Universal Screening: A Systematic Review and Meta-analysis

**DOI:** 10.1001/jamanetworkopen.2021.20736

**Published:** 2021-08-23

**Authors:** Paddy Ssentongo, Christine Hehnly, Patricia Birungi, Mikayla A. Roach, Jada Spady, Claudio Fronterre, Ming Wang, Laura E. Murray-Kolb, Laila Al-Shaar, Vernon M. Chinchilli, James R. Broach, Jessica E. Ericson, Steven J. Schiff

**Affiliations:** 1 Center for Neural Engineering, The Pennsylvania State University, University Park; 2 Department of Engineering Science and Mechanics, The Pennsylvania State University, University Park; 3 Department of Public Health Sciences, The Pennsylvania State University College of Medicine, Hershey; 4 Institute for Personalized Medicine, Department of Biochemistry and Molecular Biology, The Pennsylvania State University College of Medicine, Hershey; 5 College of Human and Health Development, The Pennsylvania State University, University Park; 6 College of Engineering, The Pennsylvania State University, University Park; 7 College of Agricultural Sciences, The Pennsylvania State University, University Park; 8 Centre for Health Informatics, Computing, and Statistics, Lancaster University, Lancaster, United Kingdom; 9 Department of Nutritional Sciences, The Pennsylvania State University, University Park; 10 Division of Pediatric Infectious Disease, The Pennsylvania State University College of Medicine, Hershey; 11 The Center for Infectious Disease Dynamics, The Pennsylvania State University, University Park; 12 Department of Neurosurgery, The Pennsylvania State University College of Medicine, Hershey; 13 Department of Physics, The Pennsylvania State University, University Park

## Abstract

**Importance:**

Congenital cytomegalovirus (cCMV) infection is the most common congenital infection and the leading acquired cause of developmental disabilities and sensorineural deafness, yet a reliable assessment of the infection burden is lacking.

**Objectives:**

To estimate the birth prevalence of cCMV in low- and middle-income countries (LMICs) and high-income countries (HICs), characterize the rate by screening methods, and delineate associated risk factors of the infection.

**Data Sources:**

MEDLINE/PubMed, Scopus, and Cochrane Database of Systematic Reviews databases were searched from January 1, 1960, to March 1, 2021, and a total of 1322 studies were identified.

**Study Selection:**

Studies that provided data on the prevalence of cCMV derived from universal screening of infants younger than 3 weeks were included. Targeted screening studies were excluded.

**Data Extraction and Synthesis:**

Preferred Reporting Items for Systematic Reviews and Meta-analyses guideline was followed. Extraction was performed independently by 3 reviewers. Quality was assessed using the Newcastle-Ottawa Scale for cohort studies. Random-effects meta-analysis was undertaken. Metaregression was conducted to evaluate the association of sociodemographic characteristics, maternal seroprevalence, population-level HIV prevalence, and screening methods with the prevalence of cCMV.

**Main Outcomes and Measures:**

Birth prevalence of cCMV ascertained through universal screening of infants younger than 3 weeks for CMV from urine, saliva, or blood samples.

**Results:**

Seventy-seven studies comprising 515 646 infants met the inclusion criteria from countries representative of each World Bank income level. The estimated pooled overall prevalence of cCMV was 0.67% (95% CI, 0.54%-0.83%). The pooled birth prevalence of cCMV was 3-fold greater in LMICs (1.42%; 95% CI, 0.97%-2.08%; n = 23 studies) than in HICs (0.48%; 95% CI, 0.40%-0.59%, n = 54 studies). Screening methods with blood samples demonstrated lower rates of cCMV than urine or saliva samples (odds ratio [OR], 0.38; 95% CI, 0.23-0.66). Higher maternal CMV seroprevalence (OR, 1.19; 95% CI, 1.11-1.28), higher population-level HIV prevalence (OR, 1.22; 95% CI, 1.05-1.40), lower socioeconomic status (OR, 3.03; 95% CI, 2.05-4.47), and younger mean maternal age (OR, 0.85; 95% CI, 0.78-0.92, older age was associated with lower rates) were associated with higher rates of cCMV.

**Conclusions and Relevance:**

In this meta-analysis, LMICs appeared to incur the most significant infection burden. Lower rates of cCMV were reported by studies using only blood or serum as a screening method.

## Key Points

**Question:**

What are the pooled prevalence of congenital cytomegalovirus infection and factors associated with the rates in high-income and low- and middle-income countries?

**Findings:**

In this systematic review and meta-analysis including 77 studies from 36 countries comprising 515 646 infants younger than 3 weeks, the pooled overall prevalence of congenital cytomegalovirus was 0.67%. The infection burden was 3-fold greater in low- and middle-income countries than in high-income countries. Lower rates were reported in screening methods using blood compared with urine or saliva.

**Meaning:**

The findings of this study suggest that low- and middle-income countries incur the greatest infection burden of congenital cytomegalovirus; a global effort to address congenital cytomegalovirus in regions with the greatest prevalence is needed to reduce disease incidence and morbidity.

## Teaser

This systematic review and meta-analysis examines the prevalence of congenital cytomegalovirus (cCMV) in young infants by economic status of countries as well as factors contributing to the diagnosis and prevalence.

## Introduction

Human herpesvirus 5, cytomegalovirus (CMV), is a common cause of asymptomatic or mild illness in immunocompetent children and adults. However, congenital CMV (cCMV) infection can lead to permanent sequelae in 15% to 18% of births, including death in 1%, neurocognitive sequelae in 5% to 15%, and hearing loss in 12% of individuals with cCMV.^1,2,3,4^

Studies have suggested that cCMV infection is a disease of disparity, with increased incidence, prevalence, and severity in low-income populations.^5,6,7,8^ However, most studies that have been used to generate estimates of the burden of cCMV infection are from high-income countries (HICs).^5,9^ Thus, the burden of cCMV infection is likely higher than currently available studies suggest. Congenital CMV is likely underreported worldwide owing to the lack of available testing in low- and middle-income countries (LMICs) and lack of systematic testing in countries of all income levels.

Differentiation of congenital vs early postnatal CMV infections requires testing near the time of birth and also after 3 weeks.^10,11^ Symptomatic infants are more likely to undergo testing shortly after birth than asymptomatic infants. However, most infants with cCMV will be asymptomatic at birth, and the sequelae of congenital infection will not be evident until weeks, months, or even years later.^12^ By the time sequelae are noted, it is too late to accurately assess the association of cCMV with the child’s outcome.^11^

These difficulties in accurately diagnosing cCMV in low-resource settings have led to estimating the burden of cCMV from prevalence estimates derived primarily in HICs.^5^ This factor, in addition to not accounting for differences in risk factors, such as maternal educational level, HIV exposure, and income distribution, contribute to underestimating the burden of infection.^5,13^ We sought to estimate the prevalence of cCMV with a meta-analysis of LMIC and HIC prevalence studies and estimate the influence that diagnostic methods as well as demographic and clinical factors have on cCMV prevalence.

## Methods

### Data Sources and Extraction

We searched the MEDLINE/PubMed, Scopus, and Cochrane Database of Systematic Reviews databases for studies published between January 1, 1960, and March 1, 2021, using a combination of medical subject headings and key words in the title and abstract denoting birth prevalence of cCMV. We used terms *CMV* or *cytomegalovirus* combined with *congenital*, or *newborn* combined with *epidemiology* (eg, *incidence*, *prevalence, burden*, *mortality*) to search peer-reviewed publications. A full list of search terms is provided in the eMethods in the Supplement. The search was performed for all countries by their categorization in the July 2019 World Bank list.^14^ This study followed the Meta-analysis of Observational Studies in Epidemiology (MOOSE) reporting guideline^15^ and the Preferred Reporting Items for Systematic Reviews and Meta-analyses (PRISMA) reporting guideline.^16^

We also searched the references cited by the retrieved articles for additional material. We applied the following inclusion criteria: (1) original peer-reviewed studies, (2) identification of cCMV through universal screening, and (3) detection of CMV based on culture or polymerase chain reaction of urine, saliva, blood, serum, or dried blood spot samples collected within 3 weeks of birth. Studies not conducted in humans, case reports, letters to editor, case series, case-control studies, comparison studies, practice guidelines, meta-analyses, literature reviews, and commentaries were excluded. Studies that did not document CMV screening methods were also excluded. We did not impose any restrictions based on language of the articles or country. To further minimize selection bias and inflation of the prevalence estimates, we excluded studies that carried out targeted screening of CMV. These included studies conducted only in the HIV population, in neonates with abnormal results on hearing screening, and those admitted to the intensive care units. Some studies used the same population in several articles. For example, articles by the CMV and Hearing Multicenter Screening study group^17,18^ had multiple overlapping populations. Therefore, we excluded articles with overlapping study populations.

Birth prevalence was defined as the number of infants with CMV infection divided by the total number of live-born infants tested for CMV in a defined population. Symptomatic cCMV infection was defined by each study, and definitions varied substantially across studies. The typical definition of the term *symptomatic* to describe clinical indications of CMV infection in newborns, known as cytomegalovirus inclusion disease, includes the presence of 1 or more of the following symptoms: petechiae, jaundice with associated hyperbilirubinemia, hepatosplenomegaly, thrombocytopenia, chorioretinitis, seizures, sensorineural hearing loss, microcephaly, intracranial calcifications, or fetal hydrops.^19,20^ The less-severe symptoms are usually transient in newborns. Many of the signs and symptoms listed are not specific to CMV or readily apparent, and hence symptomatic CMV often goes unrecognized in the absence of systematic attempts to identify it.

We extracted information on the studies’ characteristics and their participants, methods used to diagnose CMV, country-specific potential predictors of cCMV (HIV status, income level), and methodologic quality. Three of us (P.B., M.A.R., and J.S.) initially independently screened the titles and abstracts of articles and obtained the full-text articles and performed data extraction on those meeting the inclusion criteria. Three of us (P.B., M.A.R., and J.S.) jointly reviewed a random subset of articles to ensure selection accuracy. Disagreements about the included articles were resolved by 2 of us (P.S. and C.H.). A detailed account of the inclusion/exclusion process is shown in eFigure 1 in the Supplement.

Two of us (P.S. and C.H.) independently assessed the quality of the articles included in our analysis. Assessment of methodologic quality was conducted using the Newcastle-Ottawa Quality Assessment Scale, a validated tool for assessing cross-sectional, case-control, and cohort studies.^21^ Scores of 8 to the maximum score of 9 were defined as high quality; scores of 5 to 7 were defined as intermediate quality, and scores of 1 to 4 were considered low quality. Studies were included regardless of the risk of bias and quality scores, but metaregression analysis was conducted to ascertain the outcome of their inclusion.

Race or ethnicity was classified by the investigators of each study included in the meta-analysis. Options were defined by participants. Race or ethnicity was assessed because it is associated with the incidence of cCMV.

### Statistical Analysis

We adopted a narrative approach describing the number of studies, study settings, and diagnostic criteria for cCMV. Descriptive statistics are reported as proportions of a population and as medians (interquartile range).

We applied random-effects models to estimate the prevalence of cCMV and their respective 95% CIs, and we reported the pooled prevalence as a percentage of the screened newborn infants. To pool the study estimates, we used a generalized linear mixed-effects model with the logit link. We estimated all parameters via maximizing the pseudolikelihood. The generalized linear mixed-effects model method is not affected by the potential problems of back-transformation of Freeman-Tukey double arcsine transformation of single proportions.^22,23^ Individual and pooled estimates are displayed using forest plots. Between-study variation was assessed using *I*^2^, which describes the percentage of total variation across studies that is due to heterogeneity rather than chance, expressed as percentage (low [25%], moderate [50%], and high [75%]).^24^ We report the pooled estimates as percentages.

We conducted random-effects metaregression analysis to investigate the sources of heterogeneity. We examined the associations of each of the explanatory variables included in the metaregression in association with the prevalence of cCMV. These variables included study-level maternal median or mean age, the proportion with HIV/AIDS exposure, maternal CMV seroprevalence (IgG or IgM antibodies), the proportion born premature, the proportion of males, and the proportion of Black individuals for studies conducted in the US or Canada. We also explored the association of socioeconomic status (defined by country-specific income level as described by the World Bank^14^; low and middle income [≤$12 535] vs high income [≥$12 536], eFigure 2 in the Supplement), and population-level HIV prevalence. Differences in prevalence were also estimated by the biological specimen used for screening of CMV (blood, saliva, or urine). We regressed the estimates as a function of the study year to explore the prevalence trend.

To evaluate possible publication bias, we visually inspected the funnel plot for asymmetry by plotting the study effect size against SEs of the effect size and performed the Egger linear regression test^25^ and Begg rank correlation test.^26^ The Duval and Tweedie trim and fill procedure was used to adjust for the publication bias.^27^ An influence and outlier study sensitivity analysis was undertaken to estimate the association of each study with the overall pooled estimate.^28^ The metaprop, escalc, and rma functions from the R packages meta and metafor were used for the analysis.^29^ All statistical analyses were performed with R software, version 3.6.2 (R Foundation). The significance level was set at *P* < .05, and all *P* values were 2-tailed.

## Results

The initial literature search yielded 1322 articles (eFigure 1 in the Supplement); of these, we excluded 223 duplicates. After a review of titles and abstracts, we excluded 942 articles if they (1) were conducted in animals; (2) were case series, case-controls, or reviews; (3) were long-term outcomes or sequelae studies; or (4) solely compared the sensitivity of cCMV testing methods. We fully examined a total of 157 full-text articles and excluded 80 articles because (1) the study focused on a specific subpopulation (targeted screening), such as neonates with clinical signs of cCMV or a nonrepresentative demographic cohort; (2) there were overlapping study populations; and (3) the article was a systematic review. A total of 77 articles were included in this meta-analysis. The final studies were from 36 countries (eFigure 3 in the Supplement) on 5 continents and are categorized by World Health Organization regions as follows: Africa, 6 countries (6 studies)^30,31,32,33,34,35^; Americas–Latin, 5 countries (9 studies)^36,37,38,39,40,41,42,43,44^; Americas–US and Canada, 2 countries (18 studies)^17,18,45,46,47,48,49,50,51,52,53,54,55,56,57,58,59,60^; Eastern Mediterranean, 1 country (3 studies)^61,62,63^; Europe, 16 countries (24 studies)^8,64,65,66,67,68,69,70,71,72,73,74,75,76,77,78,79,80,81,82,83,84,85,86^; Southeast Asia, 2 countries (3 studies)^87,88,89^; and Western Pacific, 4 countries (14 studies).^90,91,92,93,94,95,96,97,98,99,100,101,102,103^ The present analysis included a total sample of 515 646 infants. The number of neonates included in individual studies ranged widely (minimum 117 to maximum 73 239), with a median of 2032 infants (interquartile range, 741-10 328). Details of each study included in the meta-analysis are provided in eTable 1 in the Supplement.

**Figure 1.**
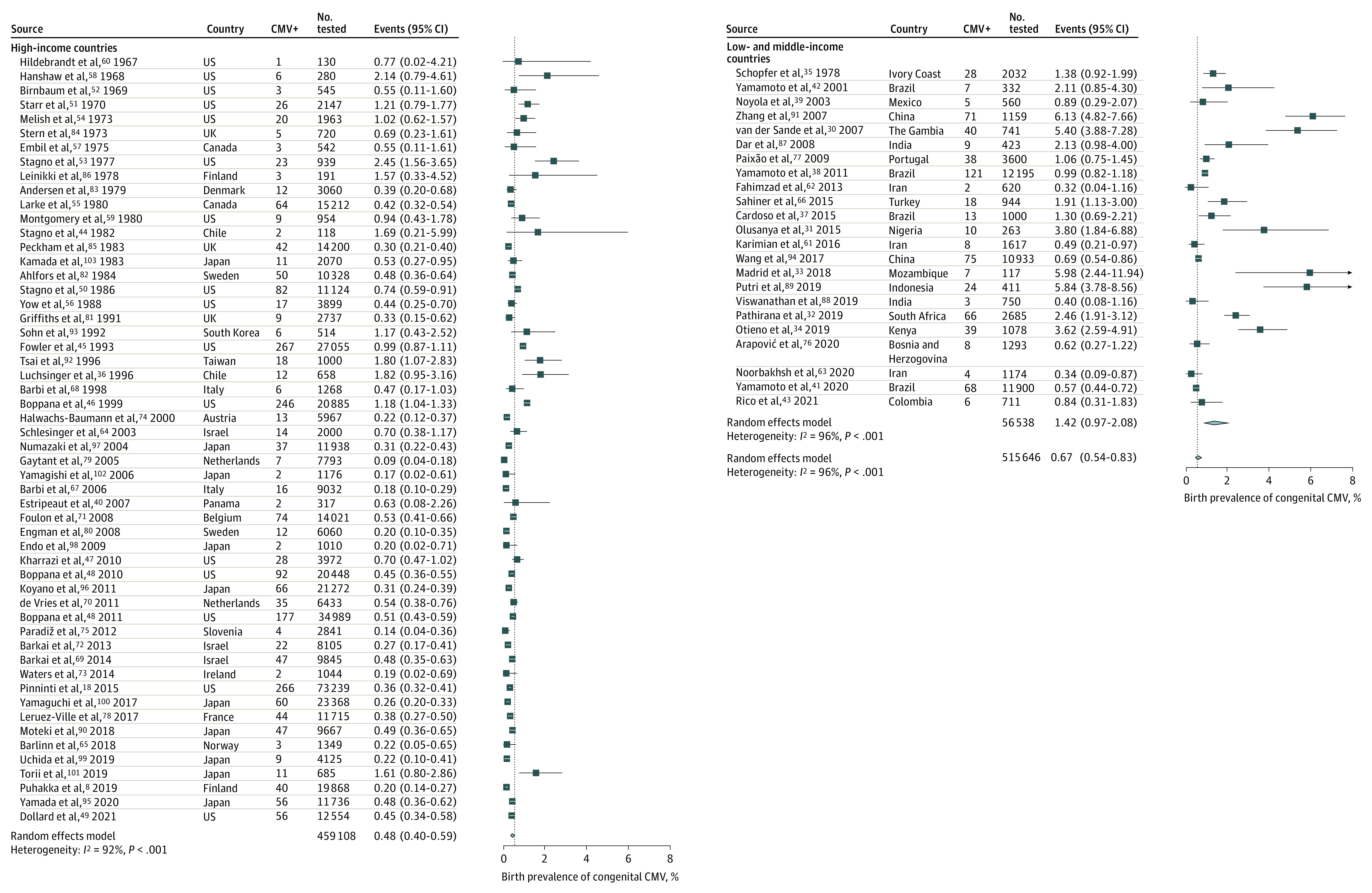
Congenital Cytomegalovirus (CMV) Prevalence by World Bank Income Level in High-Income Countries (HICs) and Low- to Middle-Income Countries Effect size values represent congenital CMV cases expressed as a percentage and their corresponding 95% CIs. Blue squares and their corresponding lines are the point estimates and 95% CIs. Diamonds represent the pooled estimate of each subgroup's prevalence (width denotes 95% CI). Heterogeneity by income level: low- to middle-income countries, *I*^2^ = 96 (23 studies); high-income countries, *I*^2^ = 92% (54 studies). Differences between subgroups were all significant at *P* < .001.

**Figure 2.**
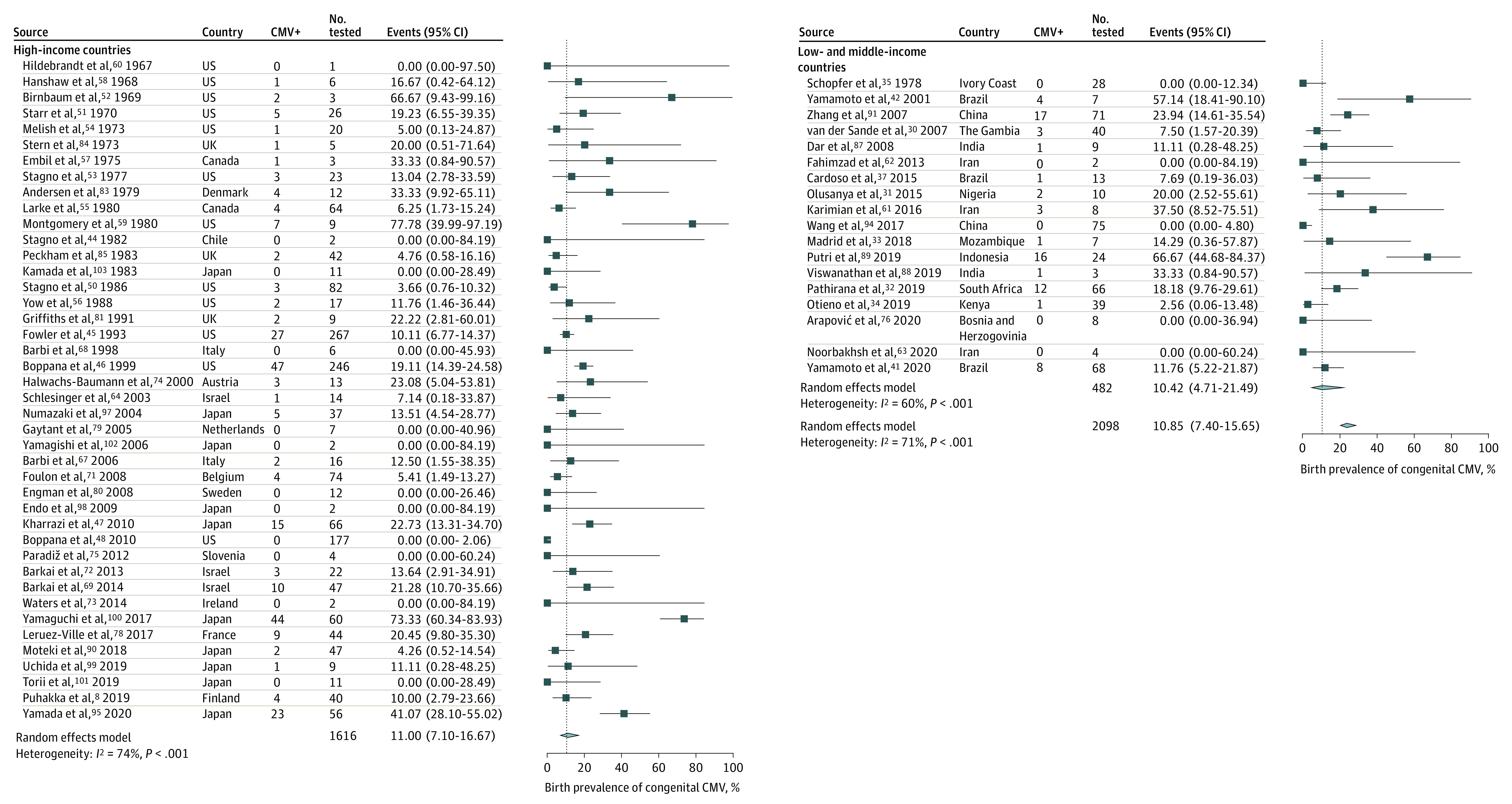
Symptomatic Congenital Cytomegalovirus (CMV) Prevalence by World Bank Income Level in High-Income Countries and Low- to Middle-Income Countries Effect size values represent congenital CMV cases expressed as a percentage and their corresponding 95% CIs. Blue squares and their corresponding lines are the point estimates and 95% CIs. Diamonds represent the pooled estimate of each subgroup's prevalence (width denotes 95% CI). Heterogeneity by income level: low- to middle-income countries (*I^2^* = 60%; heterogeneity *P* < .001; 18 studies); high-income countries (*I^2^* = 74%; heterogeneity *P* < .001; 42 studies); test for subgroup differences *P* = .90.

Consistent with the expected publication bias, source articles from HICs (54 [70%]) were encountered more frequently than those from LMICs (23 [30%]). The median methodologic quality of studies in HICs was similar to that of LMICs. The estimated pooled overall prevalence of cCMV was 0.67% (95% CI, 0.54%-0.83%). Using the random-effects model, the prevalence of cCMV was significantly higher in LMICs (1.42%; 95% CI, 0.97%-2.08%) compared with HICs (0.48%; 95% CI, 0.40%-0.59%; *P* < .001 for subgroup differences) (Figure 1). Each subgroup's heterogeneity was high, as evidenced by the *I*^2^ value (>90% in each group). The definition of symptomatic cCMV varied by study. The most common cCMV clinical signs, laboratory test results, and imaging findings reported were sensorineural hearing loss, jaundice, hepatosplenomegaly, thrombocytopenia, and central nervous system involvement (microcephaly, intracranial calcifications, enlarged ventricles) (eTable 2 in the Supplement). Sixty studies reported the proportion of symptomatic cCMV. The pooled estimate was 10.85% (95% CI, 7.40%-15.65%). No significant differences were noted in the rates of symptomatic cCMV between LMICs (10.42%; 95% CI, 4.71%-21.49%) and HICs (11.0%; 95% CI, 7.10%-16.67%). Heterogeneity by income level was 60% (*P* < .001 for heterogeneity; 18 studies) for LMICs and 74% (*P* < .001 for heterogeneity; 42 studies) for HICs (*P* = .90 for subgroup differences) (Figure 2).

Detection of CMV DNA in blood and the sensitivity of CMV polymerase chain reaction in dried blood spot samples is highly variable, ranging from 30% to 90% depending on the technique used.^17^ Therefore, screening studies based on CMV polymerase chain reaction of dried blood spots will probably underestimate cCMV prevalence, but with improved methods, this form of testing may become more useful.^49,104^ To assess possible differences in the estimates due to the biological specimens used for screening for cCMV, we conducted subgroup analysis comparing studies that carried out screening using urine and/or saliva samples with those that used only blood or serum. There was a significant difference in the cCMV prevalence rates between the biological specimens used for screening: 0.79% (95% CI, 0.63%-1.00%) for urine or saliva vs 0.31% (95% CI, 0.22%- 0.46%) for blood or serum only (*P* < .001 for subgroup differences) (eFigure 3 in the Supplement).

**Figure 3.**
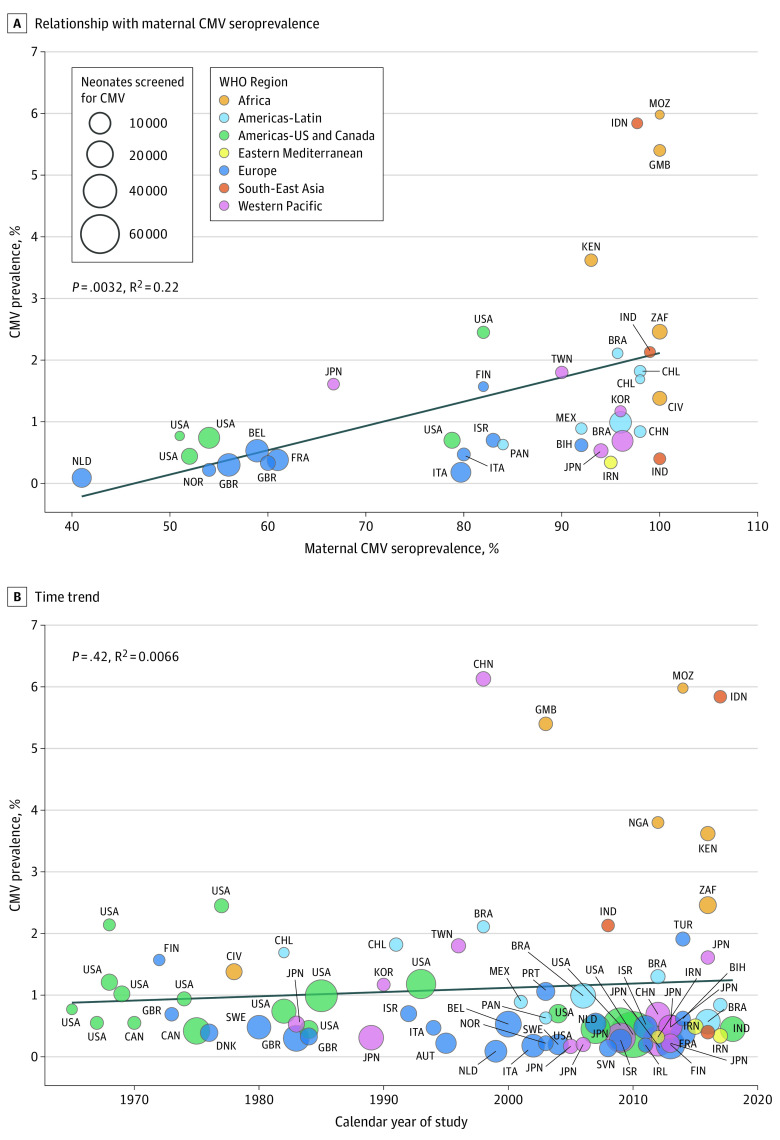
Determinants and Temporal Trends of Congenital Cytomegalovirus (cCMV) A, Maternal CMV seroprevalence is a significant determinant of congenital CMV. B, The prevalence of congenital CMV has remained consistent for the past 6 decades. Linear fit from linear regression model. Circles represent countries and are labeled by their International Organization for Standardization (ISO) code. The size of the circle is proportional to the sample size of each study and the colors represent the World Health Organization region. The ISO codes are defined in eTable 1 in the Supplement.

A univariable random-effects metaregression model revealed LMICs (odds ratio [OR], 3.03; 95% CI, 2.05-4.47), higher maternal seroprevalence (OR, 1.19; 95% CI, 1.11-1.28) (Figure 3A), higher population-level HIV prevalence (OR, 1.22; 95% CI, 1.05-1.40), and younger maternal age (OR, 0.85; 95% CI, 0.78-0.92, older age was associated with lower cCMV rates) were significant factors associated with higher cCMV prevalence (Table). Screening methods with blood samples demonstrated lower rates of cCMV than urine or saliva samples (odds ratio [OR], 0.38; 95% CI, 0.23-0.66). When the analysis was restricted to studies conducted in the US and Canada, there was an association of Black individuals with a higher risk of cCMV prevalence compared with other races (OR, 1.13; 95% CI, 1.10-1.17; *P* < .01). Temporal trend analysis indicated that cCMV prevalence rate has remained constant for the past 60 years (*R*^2^ = 0.007; *P* = .48 for temporal trend) (Figure 3B).

**Table.** Metaregression Analysis^a^

Population characteristics	No. of studies	OR (95% CI)	*P* value	*R*^2^, %^b^
Income level				
High	54	1 [Reference]		33
Low-middle	23	3.03 (2.05-4.47)	<.001^c^	
Maternal seroprevalence (per 5–percentage point increase)	37	1.19 (1.11-1.28)	<.001^c^	43
Biologic specimen tested	77			
Saliva and/or urine	63	1 [Reference]	<.001^c^	13
Blood	14	0.38 (0.23-0.66)		
Black (per 5–percentage point increase) (US and Canada studies)	7	1.13 (1.10-1.17)	<.001^c^	91
Maternal mean age (per 1-y increase from mean age of 28 y)	28	0.85 (0.78-0.92)	<.001^c^	37
Proportion male (per 5–percentage point increase)	21	1.14 (0.82-1.58)	.45	0
Study quality (per unit increase)	77	0.93 (0.78-11.00)	.39	0
Proportion premature (per 5–percentage point increase)	15	1.16 (0.98-1.37)	.08	12
Population HIV rate (per 2% increase)	23	1.22 (1.05-1.40)	.009^c^	21
Year of study (per 5-y increase)	77	0.98 (0.92-1.05)	.63	0

Abbreviation: OR, odds ratio.

^a^ Univariate metaregression using a random-effects model. The ORs and their 95% CIs are the effect sizes of the association between the contributing factors and the prevalence of congenital cytomegalovirus.

^b^ Coefficient of determination represents the amount of variation in the prevalence of congenital cytomegalovirus explained by the covariate.

^c^ Findings significant at *P* < .05.

Influence and outlier sensitivity analyses were performed for the birth prevalence of cCMV.^105^ In this analysis, 1 study was omitted and replaced 1 study at a time (leave-1-out method) from the meta-analysis, and we calculated the pooled data for the remaining studies. The pooled estimate remained close to the observed overall pooled estimate, indicating that no individual study had a large influence on the pooled estimate. The pooled point estimate ranged from 0.65 to 0.69 (eFigure 4 in the Supplement).

A symmetrical inverted funnel plot suggested the absence of publication bias (eFigure 5 in the Supplement). Similarly, neither the Begg rank correlation test (*z* = 0.83; *P* = .41) nor the Egger linear regression test (*t* = 0.05; *df*=75; *P* = .96) indicated publication bias. Nevertheless, the Duval and Tweedie trim and fill analysis was conducted to adjust for the potential small-study publication bias.^27^ Analyses suggested that the adjusted effect estimates would fall in the range of 0.57% to 0.87%, and no additional studies were added to the funnel plots (eFigure 6 in the Supplement). The median study quality score was 7 of 9 (range, 4-9).

## Discussion

We report a comprehensive systematic review and meta-analysis of cCMV epidemiologic factors. More than 1300 titles were examined to identify 77 relevant peer-reviewed publications representing more than 500 000 neonates across 36 countries. The estimated birth prevalence of cCMV was 3-fold higher in LMICs than HICs. The higher maternal CMV seroprevalence, higher population-level HIV prevalence, and young maternal age were associated with cCMV rates. Screening methods for cCMV with urine and saliva samples provided higher prevalence rates than screening methods with blood or serum samples.^17^

Others have attempted to estimate the prevalence of cCMV via systematic literature review, but these studies had limitations.^9,106^ The first systematic review of the global prevalence of cCMV was published in 2007.^9^ In this review, only 2 African countries and 1 country in the Southeast Asian region were included (Gambia and Ivory Coast from Africa and Thailand from Southeast Asia) and the risk factors were not systematically evaluated. In 2014, a systematic review of cCMV focusing on developing countries was published.^106^ Only 2 countries from Africa (Gambia and Ivory Coast) and 1 from Southeast Asia (India) were represented. This review did not evaluate the association of sociodemographic characteristics, maternal seroprevalence, fetal HIV exposure, maternal age, and race/ethnicity with the prevalence of cCMV. Since then, Africa and other LMICs have frequently published on congenital CMV (Figure 3B). Thus, an updated systematic review and meta-analysis is needed to accurately quantify the burden of cCMV to inform prevention, control, and mitigation strategies.

The large numbers of cCMV are a significant public health concern. Congenital CMV is the leading cause of nongenetic hearing loss, adding substantially to disability-adjusted life years.^1^ In addition, CMV is associated with neonatal sepsis,^7,88^ a common cause of neonatal death in the developing world, and a disorder associated with significant sequelae, such as postinfectious hydrocephalus and other complications.^107^ The current guidelines for the treatment of moderate to severe symptomatic cCMV recommends 6 months of valganciclovir.^20^ However, the safety and cost of valganciclovir limit its use in LMICs. Therefore, children most likely to have cCMV also have the least access to treatment. In addition, children in LMICs are less likely to undergo routine developmental and hearing screening and to have access to interventions should they be found deficient in either of these domains.^108^ Therefore, cCMV in the LMICs remains an important unmet public health need.

Metaregression analysis revealed that a higher prevalence of HIV infection explained a significant variation in the heterogeneity of the prevalence estimates. HIV and CMV coinfection has been shown to have a synergistic interaction contributing to the higher morbidity and mortality rates in the first year of life of those infected, compared with infants without HIV exposure.^109^ It is postulated that interaction of HIV-1 and CMV at the maternal-fetal placental interface and exposure to in utero antiretroviral therapy may cause phenotypical and functional immunologic changes in fetuses exposed to HIV, enabling increased cCMV susceptibility and burden in this population.^110^ The increased rate of cCMV in neonates exposed to HIV may be due to more-frequent CMV reinfection or reactivation in their mothers, the waning of protective immunity, or a reduced transplacental transfer of protective antibodies.^32^

Current mitigation strategies in LMICs are limited to hygienic precautions and behavioral modification among pregnant women to prevent primary infection or reinfection with a new CMV strain during pregnancy.^111^ These measures would not be expected to have substantial influences on congenital infections associated with reactivation of maternal CMV during pregnancy. We found that higher maternal seroprevalence is associated with an increased prevalence of cCMV, suggesting that nonprimary infection plays an important role in contributing to the burden of cCMV.^112,113^ Our results underscore the need for an effective vaccine and treatment strategies that can be administered in LMICs without undue risks of adverse events.^114^ Measures to facilitate universal neonatal screening to allow for early detection of sensorineural hearing loss and developmental delay are needed.^115,116,117,118,119^ A long-term neurodevelopmental delay study to assess the true impact of untreated cCMV in LMICs is needed.

### Limitations

Several limitations of the study require consideration. Africa and Southeast Asia regions were represented by relatively fewer countries (n = 8). Therefore, interpolating these few countries' estimates to the entire region introduces the potential for substantial selection and detection biases. Within- and between-country disparities in cCMV in sub-Saharan Africa have been observed. However, we used a random-effects model to represent unmodeled errors that could not be accounted for with the regression models used in this report. The very small sample sizes in some countries are another limitation that introduce selection bias and uncertainty. Furthermore, inclusions of limited populations within individual countries introduce a potential bias reflected in substantial differences in cCMV-reported prevalence. Also, symptomatic prevalence ranged widely across studies that could be linked to selection bias, and symptomatic cCMV can vary based on genetic variation of the virus that was not factored into this analysis.^120^ In addition, CMV detection methods provide varying sensitivity and specificity that could bias the pooled estimates. These limitations should serve as a road map for future studies to better estimate the global health and economic burden of cCMV and maximize capacity building for resource allocation in regions of greatest need.

## Conclusions

Congenital CMV is a major public health concern with the burden of infection estimated to be 3-fold greater in LMICs than HICs. It is necessary to better understand the economic burden of cCMV and provide more robust evaluations of health care interventions designed to reduce its incidence and impact. Nationally representative population-based studies, particularly in LMICs, are needed to assess the burden of cCMV in those regions. Region-specific prevention, diagnosis, and treatment options, and community-based education programs are needed to mitigate the incidence of cCMV and its sequelae, particularly in resource-poor settings.

## Notes

Supplement. **eMethods.** Search Terms **eTable 1.** Study Level Characteristics **eTable 2.** Clinical Signs and Symptoms of cCMV **eFigure 1.** PRISMA Diagram **eFigure 2.** Maps of WHO Regions and Income Group **eFigure 3.** Country-Specific Rates of cCMV in Countries Represented in the Current Meta-analysis **eFigure 4.** Screening Methods (Blood vs. Urine or Saliva) for cCMV **eFigure 5.** Influence and Outlier (Leave-1-Out Meta-analysis) Analysis for the Birth Prevalence of cCMV **eFigure 6.** Funnel Plots to Assess Potential for Small-Study Publication Bias **eFigure 7.** Funnel Plots From Trim and Fill Analysis
eReferences
