# Supplementary material for: Congenital Cytomegalovirus Infection Burden and Epidemiologic Risk Factors in Countries With Universal Screening: A Systematic Review and Meta-analysis
Source: JAMA Netw Open. 2021 Aug 23;4(8):e2120736. doi: 10.1001/jamanetworkopen.2021.20736 (PMC8383138; doi:10.1001/jamanetworkopen.2021.20736)
Supplement: Supplement. — eMethods. Search Terms eTable 1. Study Level Characteristics eTable 2. Clinical Signs and Symptoms of cCMV eFigure 1. PRISMA Diagram eFigure 2. Maps of WHO Regions and Income Group eFigure 3. Country-Specific Rates of cCMV in Countries Represented in the Current Meta-analysis eFigure 4. Screening Methods (Blood vs. Urine or Saliva) for cCMV eFigure 5. Influence and Outlier (Leave-1-Out Meta-analysis) Analysis for the Birth Prevalence of cCMV eFigure 6. Funnel Plots to Assess Potential for Small-Study Publication Bias eFigure 7. Funnel Plots From Trim and Fill Analysis eReferences [file jamanetwopen-e2120736-s001.pdf]

Supplementary Online Content

Ssentongo P, Hehnly C, Birungi P, et al. Congenital cytomegalovirus infection burden epidemiologic risk factors in countries with universal screening: a systematic review and meta-analysis. *JAMA Netw Open*. 2021;4(8):e2120736. doi:10.1001/jamanetworkopen.2021.20736

eMethods. Search Terms

eTable 1. Study Level Characteristics

eTable 2. Clinical Signs and Symptoms of cCMV

eFigure 1. PRISMA Diagram

eFigure 2. Maps of WHO Regions and Income Group

eFigure 3. Country-Specific Rates of cCMV in Countries Represented in the Current Meta-analysis

eFigure 4. Screening Methods (Blood vs. Urine or Saliva) for cCMV

eFigure 5. Influence and Outlier (Leave-1-Out Meta-analysis) Analysis for the Birth Prevalence of cCMV

eFigure 6. Funnel Plots to Assess Potential for Small-Study Publication Bias

eFigure 7. Funnel Plots From Trim and Fill Analysis

eReferences

This supplementary material has been provided by the authors to give readers additional information about their work.

eMethods: Search Terms

(((“Congenital” [Subheading]
AND
(“Cytomegalovirus” [MeSH]
AND
(“Africa” [MeSH] OR “Asia” [MeSH] OR “Central America” [MeSH] OR “Developing Countries” [MeSH] OR “Geographical Locations Category” [MeSH] OR “Latin America” [MeSH] OR “South America”[MeSH] OR “Dominican Republic”[tiab] OR “Principe”[tiab] OR “Puerto Rico”[tiab] OR “Sao Tome”[tiab] OR “Saudi Arabia”[tiab] OR “Sierra Leone”[tiab] OR “Virgin Islands”[tiab] OR Afghanistan*[tiab] OR Africa*[tiab] OR Albania*[tiab] OR Algeria*[tiab] OR America*[tiab] OR Andorra*[tiab] OR Angola*[tiab] OR Antarct*[tiab] OR Antigua*[tiab] OR Arab Emirate*[tiab] OR Argentin*[tiab] OR Armenia*[tiab] OR Aruba*[tiab] OR Asia*[tiab] OR Atlantic[tiab] OR Australia*[tiab] OR Austria*[tiab] OR Azerbaijan*[tiab] OR Azores Islands[tiab] OR Baham*[tiab] OR Bahra*[tiab] OR Bangladesh*[tiab] OR Barbad*[tiab] OR Barbuda*[tiab] OR Barthelemy[tiab] OR Barthélemy[tiab] OR Belarus*[tiab] OR Belgi*[tiab] OR Belize[tiab] OR Bengali[tiab] OR Benin*[tiab] OR Bermuda*[tiab] OR Bhutan*[tiab] OR Bissau[tiab] OR Bolivia*[tiab] OR Bosnia*[tiab] OR Botswana*[tiab] OR Brazil*[tiab] OR Brunei[tiab] OR Bulgaria*[tiab] OR Burkina Faso[tiab] OR Burma[tiab] OR Burmese*[tiab] OR Burundi*[tiab] OR Cabo Verd*[tiab] OR Caicos[tiab] OR Cambodia*[tiab] OR Cameroon*[tiab] OR Canad*[tiab] OR Cape Verd*[tiab] OR Cayman[tiab] OR Central[tiab] OR Chad*[tiab] OR Chile[tiab] OR China[tiab] OR Chinese[tiab] OR Colombia*[tiab] OR Comoros[tiab] OR Congo*[tiab] OR Costa Rica*[tiab] OR Cote[tiab] OR Cote d’Ivoire[tiab] OR Croatia*[tiab] OR Cuba[tiab] OR Cuban[tiab] OR Cyprus[tiab] OR Czech Republic[tiab] OR Denmark[tiab] OR developing countr*[tiab] OR developing nation*[tiab] OR Djibouti[tiab] OR Dominica*[tiab] OR East[tiab] OR East Timor[tiab] OR Ecuador*[tiab] OR Egypt*[tiab] OR El Salvador*[tiab] OR Eritrea*[tiab] OR Estonia*[tiab] OR Ethiopia*[tiab] OR Europ*[tiab] OR Fiji*[tiab] OR Finland[tiab] OR France[tiab] OR French Guiana[tiab] OR Gabon*[tiab] OR Gambia*[tiab] OR Gaza*[tiab] OR Georgia*[tiab] OR German*[tiab] OR Ghana*[tiab] OR Greece[tiab] OR Grenada*[tiab] OR Grenadines[tiab] OR Guadeloupe[tiab] OR Guatemala*[tiab] OR Guinea*[tiab] OR Guyan*[tiab] OR Haiti*[tiab] OR Herzegovina*[tiab] OR Hondura*[tiab] OR Hungary[tiab] OR Iceland*[tiab] OR income[tiab] OR India[tiab] OR Indian*[tiab] OR Indonesia*[tiab] OR Iran*[tiab] OR= Iraq*[tiab] OR Ireland[tiab] OR Israel*[tiab] OR Italian[tiab] OR Italy[tiab] OR Ivory Coast[tiab] OR Jamaica*[tiab] OR Japan*[tiab] OR Jordan*[tiab] OR Kazakh*[tiab] OR Kenya*[tiab] OR Kiribati[tiab] OR Kitts[tiab] OR Korea*[tiab] OR Kosovar*[tiab] OR Kosovo[tiab] OR Kuwait*[tiab] OR Kyrgyz*[tiab] OR Lao[tiab] OR Laos*[tiab] OR Laotian*[tiab] OR latin america[tiab] OR Latvia[tiab] OR Lebanes*[tiab] OR Lebanon[tiab] OR Lebanese[tiab] OR Lesotho[tiab] OR less developed countr*[tiab] OR less developed nation*[tiab] OR Liberia*[tiab] OR Libya*[tiab] OR Liechtenstein[tiab] OR Lithuania[tiab] OR lmic[tiab] OR lmics[tiab] OR low income countr*[tiab] OR low income nation*[tiab] OR Lucia[tiab] OR Luxembourg[tiab] OR Macedonia*[tiab] OR Madagascar*[tiab] OR Madeira Island[tiab] OR Malawi*[tiab] OR Malaysia*[tiab] OR Maldives[tiab] OR Mali[tiab] OR Malta[tiab] OR Marshall Island*[tiab] OR Martinique[tiab] OR Mauritania*[tiab] OR Mauriti*[tiab] OR Mexican*[tiab] OR Mexico[tiab] OR Micronesia*[tiab] OR middle income countr*[tiab] OR middle income nation*[tiab] OR Moldova[tiab] OR Moldova*[tiab] OR Monaco[tiab] OR Mongolia*[tiab] OR Montenegr*[tiab] OR Montserrat[tiab] OR Morocc*[tiab] OR Mozambique[tiab] OR Myanmar[tiab] OR Namibia*[tiab] OR Nauru[tiab] OR Nepal*[tiab] OR Nevis[tiab] OR New Zealand[tiab] OR Nicaragua*[tiab] OR Niger*[tiab] OR Nigeria*[tiab] OR North[tiab] OR Norway[tiab] OR Oman*[tiab] OR Pacific[tiab] OR Pakistan*[tiab] OR Palau[tiab] OR Palestin*[tiab] OR Panama*[tiab] OR Papua[tiab] OR Paraguay*[tiab] OR Peru*[tiab] OR Philippin*[tiab] OR Poland[tiab] OR poor countr*[tiab] OR poor nation*[tiab] OR Portug*[tiab] OR Principe[tiab] OR Qatar*[tiab] OR Romania*[tiab] OR Russia*[tiab] OR Rwanda*[tiab] OR Saint Lucia[tiab] OR Saint Vincent[tiab] OR Samoa*[tiab] OR San Marino[tiab] OR Sao Tome[tiab] OR Senegal*[tiab] OR Serbia*[tiab] OR Seychelles[tiab] OR Sierra Leone*[tiab] OR Singapore[tiab] OR Slovakia*[tiab] OR Slovenia*[tiab] OR Solomon[tiab] OR Solomon Island*[tiab] OR Somalia*[tiab] OR South [tiab] OR Spain[tiab] OR Sri Lanka[tiab] OR Sudan*[tiab] OR Suriname*[tiab] OR Swaziland*[tiab] OR Swed*[tiab] OR Switzerland[tiab] OR Syria*[tiab] OR Taiwan[tiab] OR Tajik*[tiab] OR Tanzania*[tiab] OR Thai*[tiab] OR third world countr*[tiab] OR third world nation*[tiab] OR Timor Leste[tiab] OR Timor*[tiab] OR Tobago[tiab] OR Togo*[tiab] OR Tonga*[tiab] OR Trinidad*[tiab] OR Tunisia*[tiab] OR Turkey[tiab] OR Turkish[tiab] OR Turkmen*[tiab] OR Turks[tiab] OR Tuvalu*[tiab] OR Uganda*[tiab] OR Ukrain*[tiab] OR under developed countr*[tiab] OR underdeveloped nation*[tiab] OR underdeveloped nation*[tiab] OR underdeveloped nation*[tiab] OR United Kingdom[tiab] OR United States[tiab] OR Uruguay[tiab] OR Uzbeki*[tiab] OR Vanuatu*[tiab] OR Vatican[tiab] OR Venezuela*[tiab] OR Viet nam*[tiab] OR Vietnam*[tiab] OR Vincent[tiab] OR West[tiab] OR West Bank[tiab] OR Yemen*[tiab] OR Zambia*[tiab] OR Zimbabw*[tiab])
NOT
(“Animals”[MeSH]

© 2021 Ssentongo P et al. *JAMA Network Open.*

eTable 1. Study Level Characteristics

First Author	Publication year	Study Period	Country	ISO code	Screening Biological Specimen	Laboratory Method	Tested	Positive	Symptomatic (%)	Male (%)	Maternal age (Y)	Maternal Seroprevalence (%)	Study Quality (NOS)
Tsai	1996		Taiwan	TWN	Urine	Culture, PCR	1000	18		72		90	8
Sohn	1992	1989-1991	South Korea	KOR	Urine and cord blood	Culture	514	6			30	96	8
Yamada	2020	2009-2018	Japan	JPN	Urine	PCR	11736	56	41				7
Koyano	2011	2008-2010	Japan	JPN	Urine	PCR	21272	66	23		30		7
Numazaki	2004	1977-2002	Japan	JPN	Urine	Culture	11938	37	14	54			7
Yamaguchi	2017	2008-2015	Japan	JPN	Urine	PCR	23368	60	73				7
Endo	2009	2005-2007	Japan	JPN	Blood	PCR	1010	2	0	51	30		8
Uchida	2019	2009-2017	Japan	JPN	Urine	PCR	4125	9	11	52	30		8
Moteki	2018	2011-2016	Japan	JPN	DBS	PCR	9667	47	4	64			8
Torii	2019	2014-2017	Japan	JPN	Urine	PCR	685	11	0		31	67	9
Yamagishi	2006	2004-2005	Japan	JPN	Blood	PCR	1176	2	0				6
Zhang	2007	1997-2000	China	CHN	Urine	PCR	1159	71	24	54			7
Wang	2017	2011-2013	China	CHN	DBS, Saliva	PCR	10933	75	0			96	7
Putri	2019	2016-2017	Indonesia	IDN	Saliva, Urine	PCR	411	24	67			98	8
Dar	2008	2008	India	IND	Saliva, Urine	PCR	423	9	11		22	99	8
Viswanathan	2019	2015-2017	India	IND	Saliva, Urine, Blood	PCR	750	3	33	50	29	100	9
Sahiner	2015	2013-2014	Turkey	TUR	Saliva, Urine	PCR	944	18					6
Paradiz	2012	2007-2008	Slovenia	SVN	Urine, Blood	PCR, Culture	2841	4	0	50			8
Paixão	2009	2003-2004	Portugal	PRT	Blood	PCR	3600	38					6
Barlinn	2018	1999-2008	Norway	NOR	Blood	PCR	1349	3			30	54	9
Gayant	2005	1998-2000	Netherlands	NLD	Blood, Throat swab, Urine	Serology, PCR, Culture	7793	7	0			41	8
de Vries	2011	2007	Netherlands	NLD	Blood	PCR	6433	35					9
Barbi	1998	1994-1995	Italy	ITA	Saliva, Urine, Blood	PCR	1268	6	0			80	8
Barbi	2006	2002-2003	Italy	ITA	DBS	PCR	9032	16	13	53	32	80	9
Schlesinger	2003	1992-1993	Israel	ISR	Urine	PCR, Culture	2000	14	7	71		83	9
Barkai	2014	2011-2012	Israel	ISR	Saliva, Urine (for confirmation)	PCR, Culture	9845	47	21	67	31		8

© 2021 Ssentongo P et al. *JAMA Network Open.*

Barkai	2013	2009-2010	Israel	ISR	Infant cord blood, Urine	PCR, Culture	8105	22	14	50	30		8
Waters	2014	2011-2012	Ireland	IRL	Urine, Saliva	PCR	1044	2	0				8
Puhakka	2019	2012-2015	Finland	FIN	Saliva	PCR	19868	40	10		32		8
Arapovic	2020	2010-2019	Bosnia and Herzegovina	BIH	Blood, Saliva	PCR	1293	8	0		30	92	9
Foulon	2008	1996-2006	Belgium	BEL	Urine	Culture	14021	74	5			59	8
Halwachs-Baumann	2000	1993-1997	Austria	AUT	Urine, Blood	Serology, PCR, Culture	5967	13	23				9
Leruez-Ville	2017	2013-2015	France	FRA	Saliva	PCR	11715	44	20		32	61	8
Engman	2008	2003-2004	Sweden	SWE	Blood, Urine	PCR, Culture	6060	12	0				7
Karimian	2016	2014-2016	Iran	IRN	Urine	PCR	1617	8	38	57	27		9
Fahimzad	2013	2012	Iran	IRN	Saliva	PCR	620	2	0	53			9
Noorbakhsh	2020	2017	Iran	IRN	Blood	PCR	1174	4	0	57	28	95	8
Fowler	1993	1980-1990	USA	USA	Urine	Culture	27055	267	10				8
Boppana	1999	1991-1997	USA	USA	Urine, Saliva	DEAFF	20885	246	19				9
Kharrazi	2010	2004-2005	USA	USA	Blood	PCR	3972	28				79	8
Boppana	2010	2007-2008	USA	USA	DBS, Saliva	PCR, rapid Culture	20448	92		51	27		9
Pinninti	2016	2008-2012	USA	USA	Saliva, Urine	PCR, rapid Culture	73239	266					8
Boppana	2011	2008-2009	USA	USA	Saliva, Urine	PCR, rapid Culture	34989	177	0	51	27		9
Dollard	2021	2016-2019	USA	USA	Saliva, DBS, Urine	PCR	12554	56					9
Estripeaut	2007	2003-2004	Panama	PAN	Urine	PCR	317	2				84	7
Noyola	2003	2001	Mexico	MEX	Saliva	Culture	560	5				92	6
Luschsinger	1996	1989-1994	Chile	CHL	urine and saliva (throatswab)‡	Culture, PCR	658	12				98	4
Yamamoto	2001	1998-1999	Brazil	BRA	Urine, Blood	PCR, Culture	332	7	57			96	6
Cardoso	2015	2012	Brazil	BRA	Urine, Saliva	PCR	1000	13	8				8
Yamamoto	2011	2003-2009	Brazil	BRA	Urine, Saliva	PCR, culture	12195	121				96	9
Yamamoto	2020	2013-2017	Brazil	BRA	Urine, Saliva	PCR	11900	68	12	52	26		9
Rico	2021	2017-2018	Colombia	COL	Urine	PCR	711	6				98	7
Pathirana	2019	2016	South Africa	ZAF	Saliva, Urine	PCR	2685	66	18	48	28	100	8
Olusanya	2015	2012-2013	Nigeria	NGA	Saliva	PCR	263	10	20		30		9

Madrid	2018	2014-2015	Mozambique	MOZ	Blood, Nasopharyngeal swab	PCR	117	7	14		22	100	8
van der Sande	2007	2002-2005	The Gambia	GMB	Urine	PCR	741	40	8	53		100	9
Otieno	2019	2015-2017	Kenya	KEN	Saliva, DBS	PCR	1078	39	3			93	8
Griffiths	1991	1983-1985	United Kingdom	GBR	Urine	Culture	2737	9	22			60	7
Stagno	1986	1978-1984	USA	USA	Urine	Culture	11124	82	4		27	54	8
Starr	1970	1967-1968	USA	USA	Urine	Culture	2147	26	19		23		6
Birnbaum	1969	1966-1967	USA	USA	Urine	Culture	545	3	67				5
Ahlfors	1984	1977-1982	Sweden	SWE	Urine	Culture	10328	50					6
Andersen	1979	1974-1977	Denmark	DNK	Urine	Culture	3060	12	33				7
Stagno	1977		USA	USA	Urine	Culture	939	23	13		22	82	8
Melish†	1973	1968-1970	USA	USA	Urine	Culture	1963	20	5				7
Stern	1973		United Kingdom	GBR	Urine	Culture	720	5	20				6
Peckham‡	1983	1979-1982	United Kingdom	GBR	Throat swab	Culture	14200	42	5			56	7
Larke	1980	1974-1975	Canada	CAN	Urine	Culture	15212	64	6				6
Schopfer	1978		Ivory Coast	CIV	Urine	Culture	2032	28	0			100	5
Stagno	1982		Chile	CHL	Urine	Culture	118	2	0		23	98	8
Yow	1988	1981-1986	USA	USA	Urine	Culture	3899	17	12		29	52	8
Embil	1975	1969-1971	Canada	CAN	Urine	Culture	542	3	33	56			7
Kamada	1983		Japan	JPN	Urine	Culture	2070	11	0			94	5
Hanshaw	1968	1967-1968	USA	USA	Urine	Culture	280	6	17				4
Montgomery	1980	1972-1975	USA	USA	Urine	Culture	954	9	78		20		8
Hildebrandt	1967	1964-1965	USA	USA	Urine	Culture	130	1	0			51	5
Leikikki	1978	1972	Finland	FIN	Urine	Culture	191	3				82	7

PCR: polymerase chain reaction; NOS: Newcastle-Ottawa Quality Assessment Scale; DBS: dry blood spot; DEAFF: early antigen fluorescent foci; ISO, International Organization for Standardization.

Footnote

†only urine screening results reported in the meta-analysis; ‡Throat swab proxy for saliva

© 2021 Ssentongo P et al. *JAMA Network Open.*

eTable 2: Clinical signs and symptoms of cCMV. Frequency with percentages of clinical signs of cCMV reported in the studies included in the meta-analysis.

**Clinical signs and symptoms of cCMV**	**Frequency (n/number of studies)**
jaundice	15/27 (56%)
elevated liver enzymes	3/27 (11%)
hepatosplenomegaly	13/27 (48%)
thrombocytopenia	16/27 (59%)
petechiae	9/27 (33%)
anemia	4/27 (15%)
seizures	4/27 (15%)
chorioretinitis	8/27 (30%)
sensorineural hearing loss (deafness)	16/27 (59%)
CNS involvement (microcephaly, intracranial calcifications, enlarged ventricles)	16/27 (59%)
sepsis	3/27 (11%)

© 2021 Ssentongo P et al. *JAMA Network Open.*

eFigure 1. PRISMA Diagram

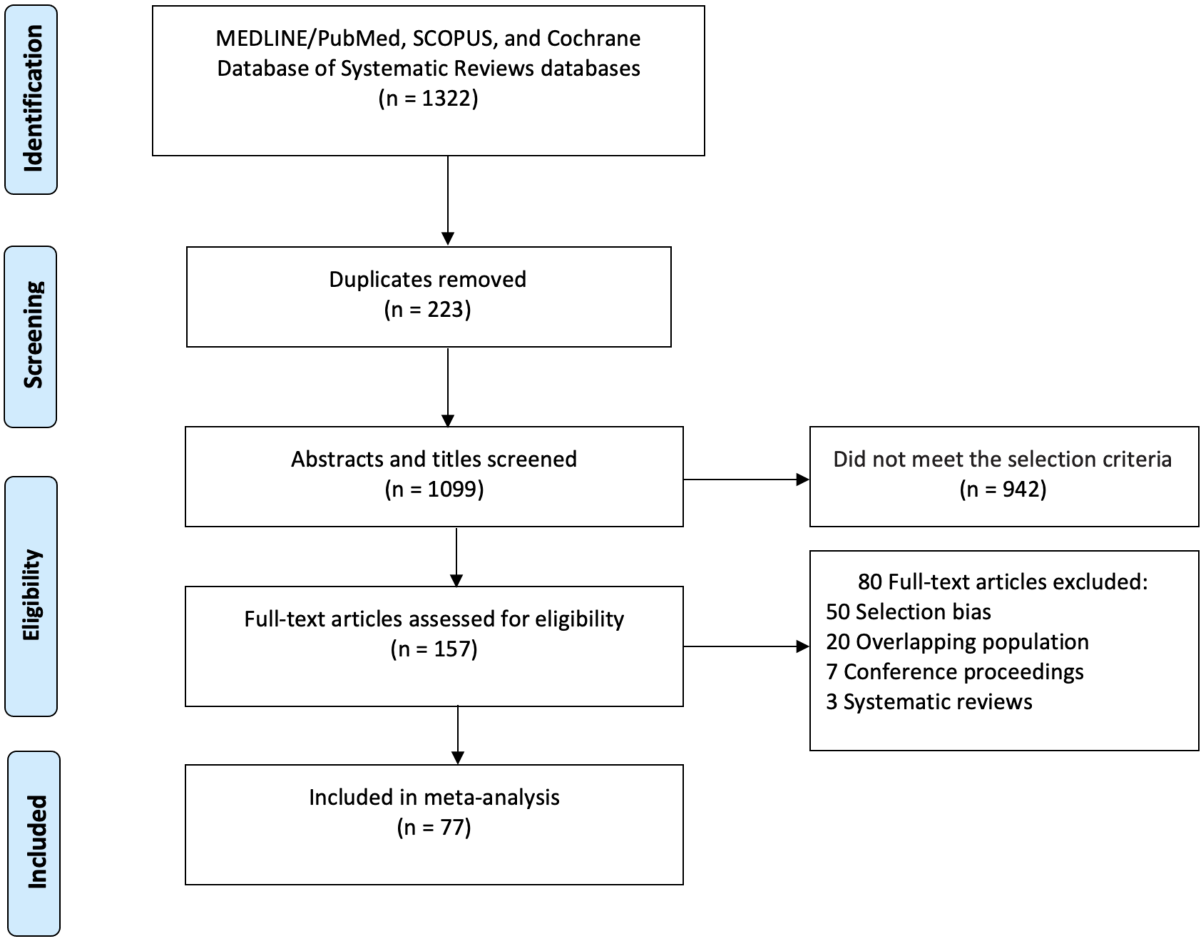

© 2021 Ssentongo P et al. *JAMA Network Open.*

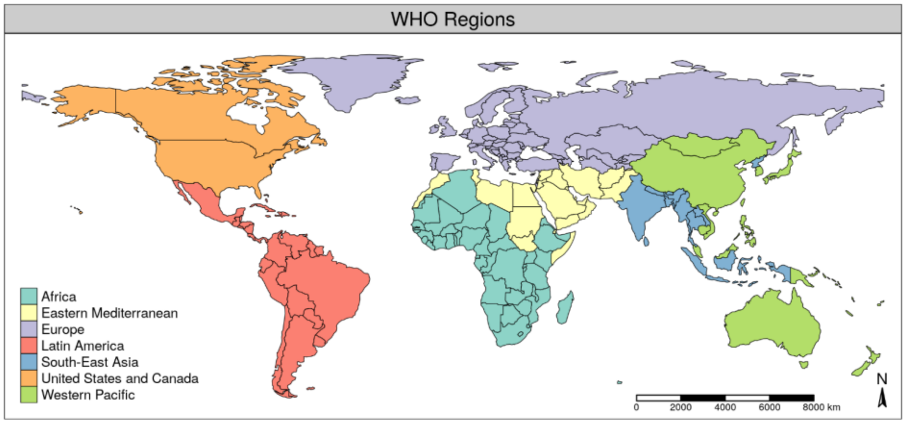

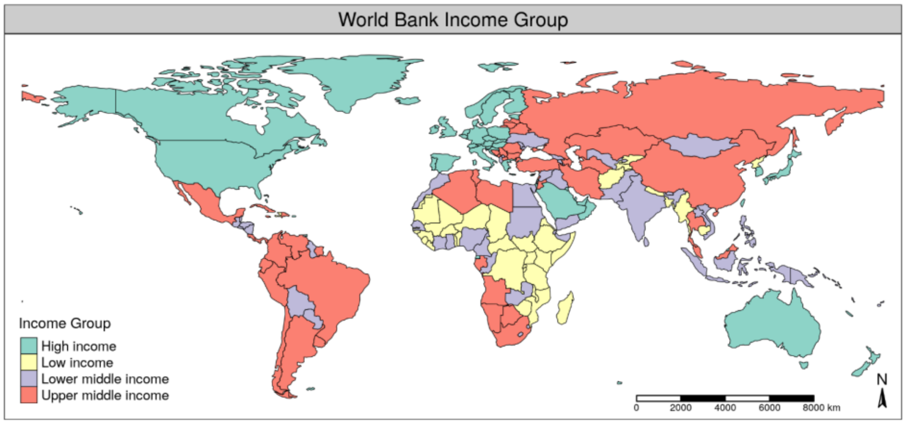

eFigure 2: Maps of WHO regions and income group. Maps were produced with the R software for statistical computing version 3.6.3. The world shapefile was retrieved from the **spData** package (version 0.3.0, https://CRAN.R-project.org/package=spData) and the maps created with the **tmap** package (version 3.2.2, https://CRAN.R-project.org/package=tmap).

© 2021 Ssentongo P et al. *JAMA Network Open.*

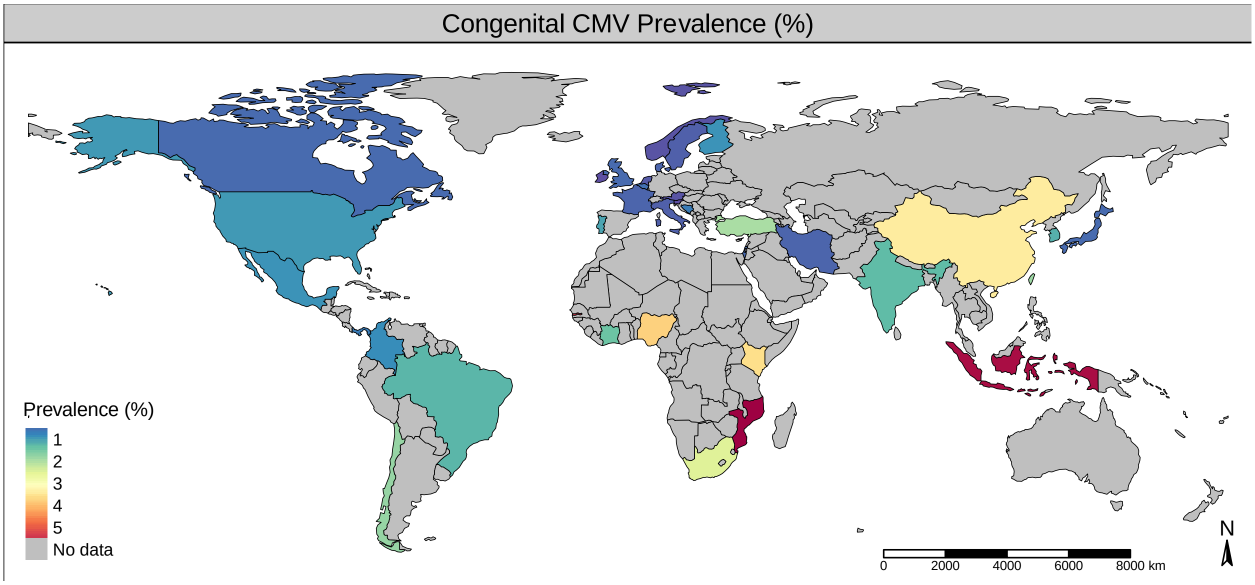

eFigure 3: Country-specific rates of cCMV in countries represented in the current meta-analysis. Birth prevalence of cCMV (%) by country. Countries with more than one study, the weighted rate was obtained using random effects models. Maps were produced with the R software for statistical computing version 3.6.3. The world shapefile was retrieved from the **spData** package (version 0.3.0, https://CRAN.R-project.org/package=spData) and the maps created with the **tmap** package (version 3.2.2, https://CRAN.R-project.org/package=tmap).

© 2021 Ssentongo P et al. *JAMA Network Open.*

Author, Year of publication, Country	CMV+	Number tested	Events	95%−CI
**Urine or Saliva**				
Hildebrandt, 1967, USA	1	130	0.77	[0.02; 4.21]
Hanshaw, 1968, USA	6	280	2.14	[0.79; 4.61]
Birnbaum, 1969, USA	3	545	0.55	[0.11; 1.60]
Starr, 1970, USA	26	2147	1.21	[0.79; 1.77]
Melish, 1973, USA	20	1963	1.02	[0.62; 1.57]
Stern, 1973, United Kingdom	5	720	0.69	[0.23; 1.61]
Embil, 1975, Canada	3	542	0.55	[0.11; 1.61]
Stagno, 1977, USA	23	939	2.45	[1.56; 3.65]
Schopfer, 1978, Ivory Coast	28	2032	1.38	[0.92; 1.99]
Leikikki, 1978, Finland	3	191	1.57	[0.33; 4.52]
Andersen, 1979, Denmark	12	3060	0.39	[0.20; 0.68]
Larke, 1980, Canada	64	15212	0.42	[0.32; 0.54]
Montgomery, 1980, USA	9	954	0.94	[0.43; 1.78]
Stagno, 1982, Chile	2	118	1.69	[0.21; 5.99]
Peckham, 1983, United Kingdom	42	14200	0.30	[0.21; 0.40]
Kamada, 1983, Japan	11	2070	0.53	[0.27; 0.95]
Ahlfors, 1984, Sweden	50	10328	0.48	[0.36; 0.64]
Stagno, 1986, USA	82	11124	0.74	[0.59; 0.91]
Yow, 1988, USA	17	3899	0.44	[0.25; 0.70]
Griffiths, 1991, United Kingdom	9	2737	0.33	[0.15; 0.62]
Sohn, 1992, South Korea	6	514	1.17	[0.43; 2.52]
Fowler, 1993, USA	267	27055	0.99	[0.87; 1.11]
Tsai, 1996, Taiwan	18	1000	1.80	[1.07; 2.83]
Luschsinger, 1996, Chile	12	658	1.82	[0.95; 3.16]
Barbi, 1998, Italy	6	1268	0.47	[0.17; 1.03]
Boppana, 1999, USA	246	20885	1.18	[1.04; 1.33]
Yamamoto, 2001, Brazil	7	332	2.11	[0.85; 4.30]
Schlesinger, 2003, Israel	14	2000	0.70	[0.38; 1.17]
Noyola, 2003, Mexico	5	560	0.89	[0.29; 2.07]
Numazaki, 2004, Japan	37	11938	0.31	[0.22; 0.43]
Zhang, 2007, China	71	1159	6.13	[4.82; 7.66]
Estripeaut, 2007, Panama	2	317	0.63	[0.08; 2.26]
van der Sande, 2007, The Gambia	40	741	5.40	[3.88; 7.28]
Dar, 2008, India	9	423	2.13	[0.98; 4.00]
Foulon, 2008, Belgium	74	14021	0.53	[0.41; 0.66]
Boppana, 2010, USA	92	20448	0.45	[0.36; 0.55]
Koyano, 2011, Japan	66	21272	0.31	[0.24; 0.39]
Boppana, 2011, USA	177	34989	0.51	[0.43; 0.59]
Yamamoto, 2011, Brazil	121	12195	0.99	[0.82; 1.18]
Paradiz, 2012, Slovenia	4	2841	0.14	[0.04; 0.36]
Fahimzad, 2013, Iran	2	620	0.32	[0.04; 1.16]
Barkai, 2014, Israel	47	9845	0.48	[0.35; 0.63]
Waters, 2014, Ireland	2	1044	0.19	[0.02; 0.69]
Sahiner, 2015, Turkey	18	944	1.91	[1.13; 3.00]
Cardoso, 2015, Brazil	13	1000	1.30	[0.69; 2.21]
Olusanya, 2015, Nigeria	10	263	3.80	[1.84; 6.88]
Karimian, 2016, Iran	8	1617	0.49	[0.21; 0.97]
Pinninti, 2016, USA	266	73239	0.36	[0.32; 0.41]
Yamaguchi, 2017, Japan	60	23368	0.26	[0.20; 0.33]
Wang, 2017, China	75	10933	0.69	[0.54; 0.86]
Leruez−Ville, 2017, France	44	11715	0.38	[0.27; 0.50]
Madrid, 2018, Mozambique	7	117	5.98	[2.44; 11.94]
Uchida, 2019, Japan	9	4125	0.22	[0.10; 0.41]
Torii, 2019, Japan	11	685	1.61	[0.80; 2.86]
Putri, 2019, Indonesia	24	411	5.84	[3.78; 8.56]
Viswanathan, 2019, India	3	750	0.40	[0.08; 1.16]
Puhakka, 2019, Finland	40	19868	0.20	[0.14; 0.27]
Pathirana, 2019, South Africa	66	2685	2.46	[1.91; 3.12]
Otieno, 2019, Kenya	39	1078	3.62	[2.59; 4.91]
Yamada, 2020, Japan	56	11736	0.48	[0.36; 0.62]
Yamamoto, 2020, Brazil	68	11900	0.57	[0.44; 0.72]
Dollard, 2021, USA	56	12554	0.45	[0.34; 0.58]
Rico, 2021, Colombia	6	711	0.84	[0.31; 1.83]
**Random effects model**		**449015**	**0.79**	**[0.63; 1.00]**
Heterogeneity: $I^2 = 96\%$, $p < 0.0001$				
**Blood**				
Halwachs−Baumann, 2000, Austria	13	5967	0.22	[0.12; 0.37]
Gayant, 2005, Netherlands	7	7793	0.09	[0.04; 0.18]
Yamagishi, 2006, Japan	2	1176	0.17	[0.02; 0.61]
Barbi, 2006, Italy	16	9032	0.18	[0.10; 0.29]
Engman, 2008, Sweden	12	6060	0.20	[0.10; 0.35]
Endo, 2009, Japan	2	1010	0.20	[0.02; 0.71]
Paixão, 2009, Portugal	38	3600	1.06	[0.75; 1.45]
Kharrazi, 2010, USA	28	3972	0.70	[0.47; 1.02]
de Vries, 2011, Netherlands	35	6433	0.54	[0.38; 0.76]
Barkai, 2013, Israel	22	8105	0.27	[0.17; 0.41]
Moteki, 2018, Japan	47	9667	0.49	[0.36; 0.65]
Barlinn, 2018, Norway	3	1349	0.22	[0.05; 0.65]
Arapovic, 2020, Bosnia and Herzegovina	8	1293	0.62	[0.27; 1.22]
Noorbakhsh, 2020, Iran	4	1174	0.34	[0.09; 0.87]
**Random effects model**		**66631**	**0.31**	**[0.22; 0.46]**
Heterogeneity: $I^2 = 86\%$, $p < 0.0001$				
**Random effects model**		**515646**	**0.67**	**[0.54; 0.83]**
Heterogeneity: $I^2 = 96\%$, $p < 0.0001$				

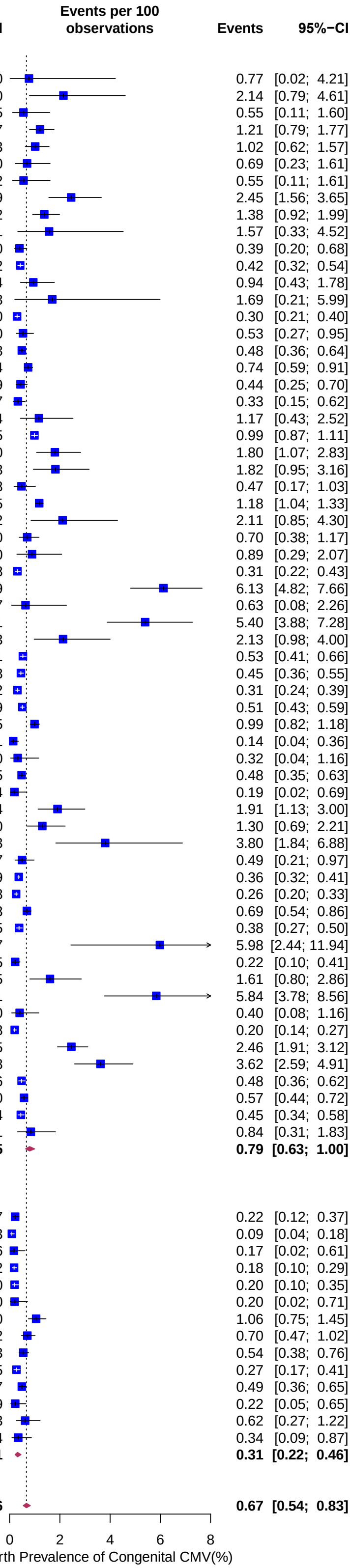

© 2021 Ssentongo P et al. *JAMA Network Open.*

eFigure 4: Screening methods (Blood vs. Urine or Saliva) for cCMV. Forest plot of cCMV rates by screen methods (Blood only Vs. Urine/Saliva). Effect size values represent congenital CMV cases expressed as a percentage and their corresponding 95% CI. Blue squares and their corresponding lines are the point estimates and 95% confidence intervals (95% CI). Maroon diamonds represent the pooled estimate of each subgroup's incidence (width denotes 95% CI). Heterogeneity by screening method: Urine or saliva (I^2 = 96%, p for heterogeneity < 0.0001; 63 studies); HICs (I^2 = 86%, p for heterogeneity < 0.0001; 14 studies); p for comparing the subgroups <0.0001.

© 2021 Ssentongo P et al. *JAMA Network Open.*

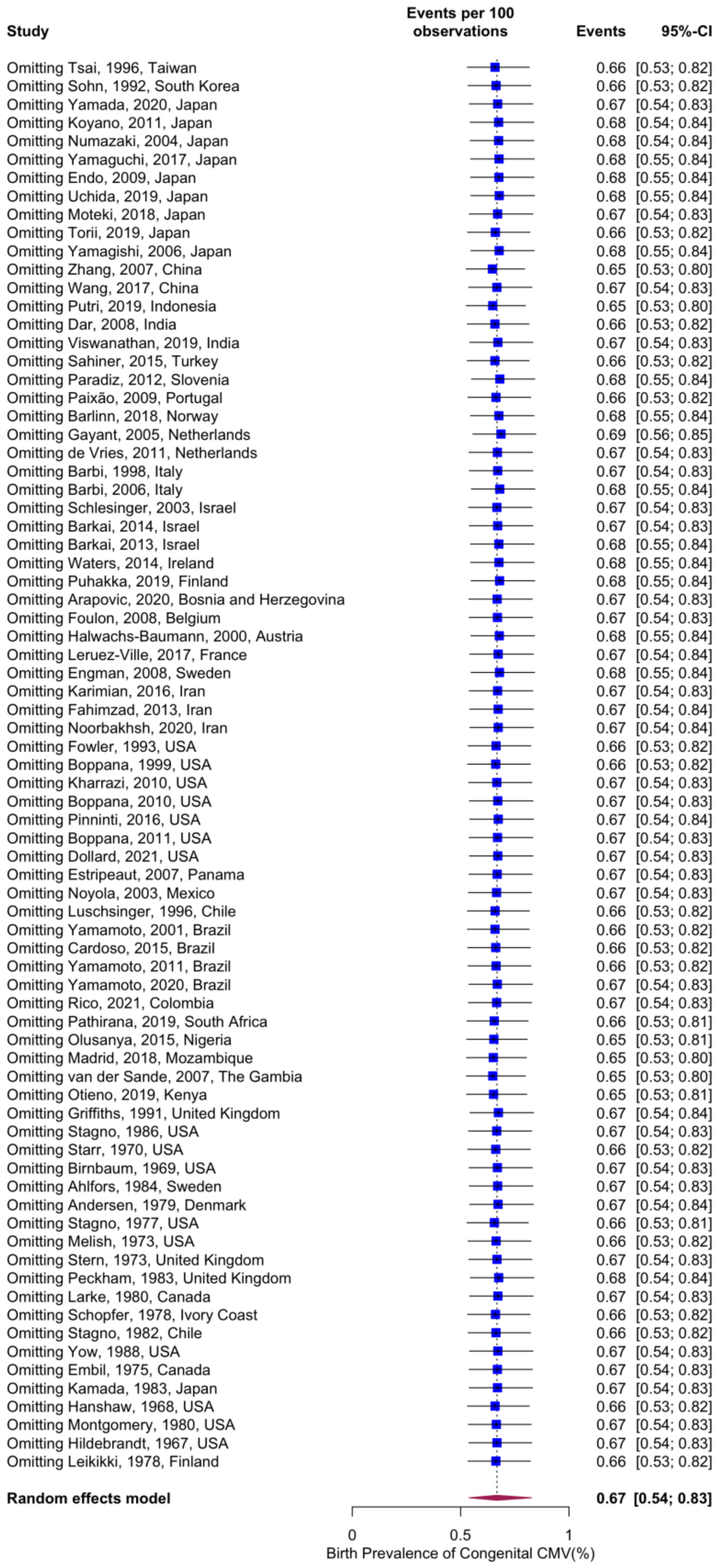

© 2021 Ssentongo P et al. *JAMA Network Open.*

eFigure 5: Influence and outlier (leave-one-out meta-analysis) analysis for the birth prevalence of cCMV.[1] The results of our outlier and influence analysis show the recalculated pooled point estimate ranged from 0.65% to 0.69% when one study omitted each time.

© 2021 Ssentongo P et al. *JAMA Network Open.*

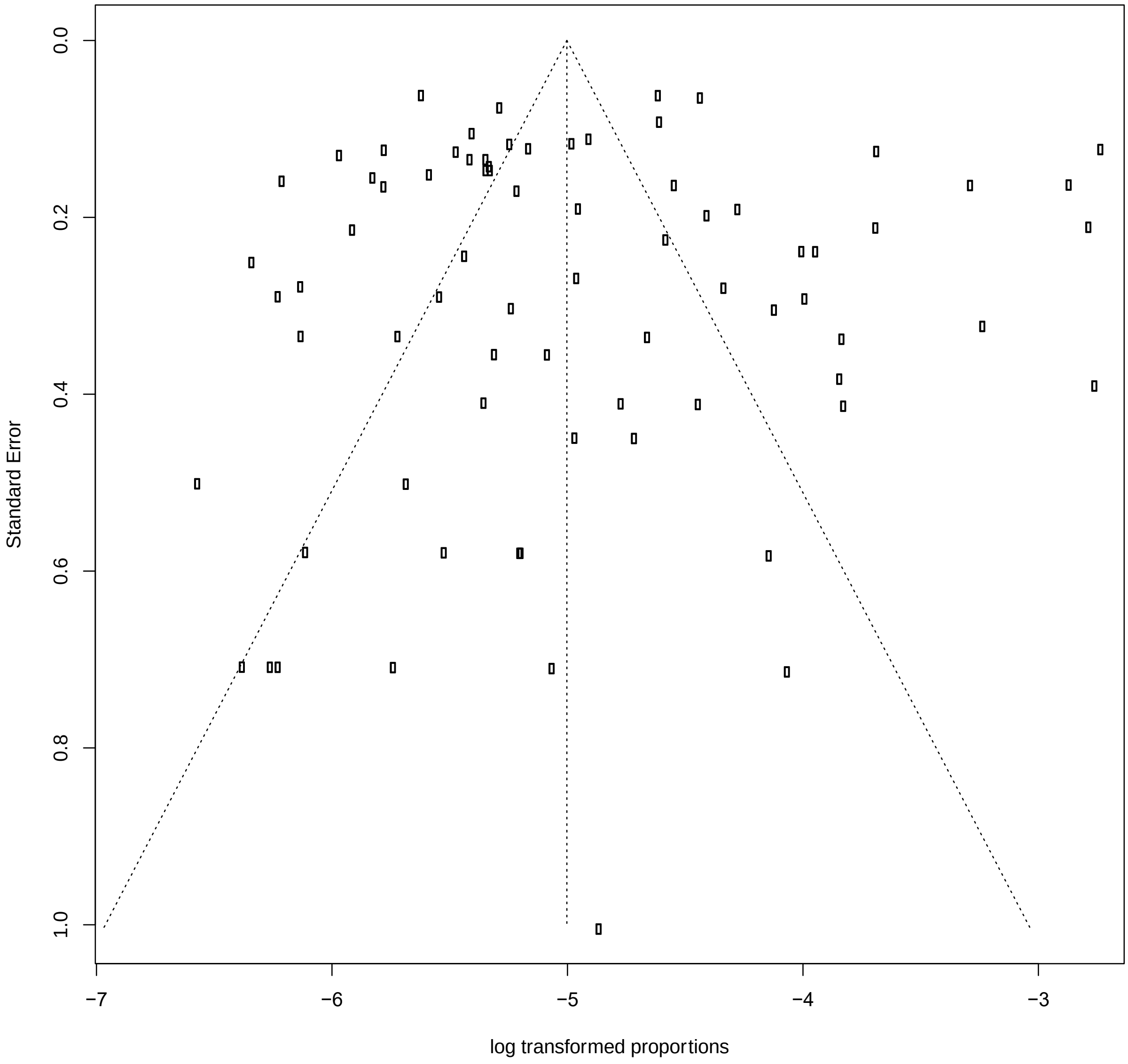

eFigure 6: Funnel plots to assess potential for small-study publication bias.[2] Symmetrical inverted funnel plot suggested absence of publication bias.

© 2021 Ssentongo P et al. *JAMA Network Open.*

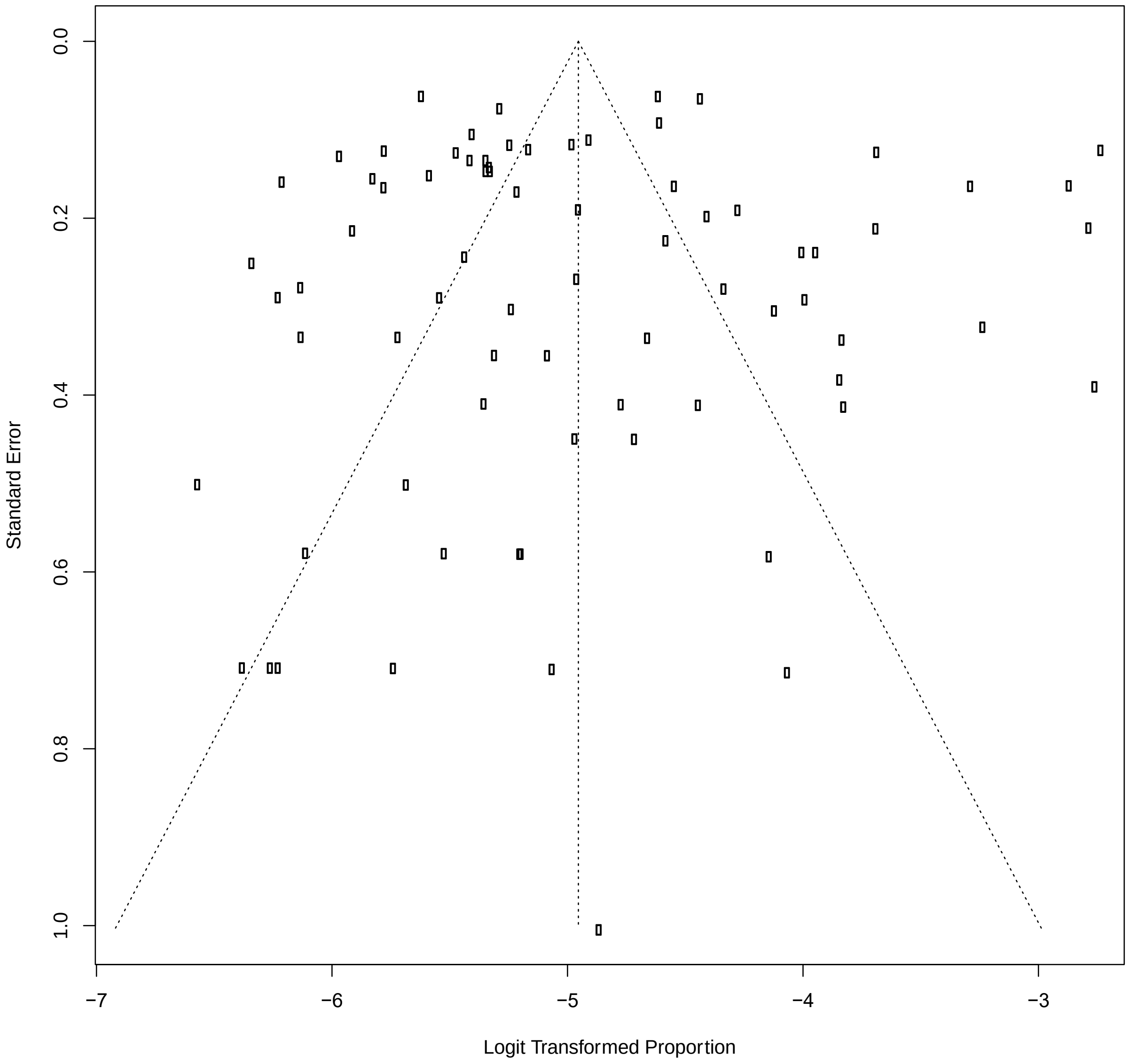

eFigure 7: Funnel plots from trim and fill analysis. Duval & Tweedie trim and fill analytical method suggests that the adjusted effect estimates would fall in the range of 0.57% to 0.87%, and no additional studies were added.[3]

© 2021 Ssentongo P et al. *JAMA Network Open.*

eReferences

1. Patsopoulos NA, Evangelou E, Ioannidis JP. Sensitivity of between-study heterogeneity in meta-analysis: proposed metrics and empirical evaluation. *International journal of epidemiology.* 2008;37(5):1148-1157.
2. Sterne JA, Becker BJ, Egger M. The funnel plot. *Publication bias in meta-analysis: Prevention, assessment and adjustments.* 2005:75-98.
3. Duval S, Tweedie R. Trim and fill: a simple funnel-plot–based method of testing and adjusting for publication bias in meta-analysis. *Biometrics.* 2000;56(2):455-463.

© 2021 Ssentongo P et al. *JAMA Network Open.*
